# Myeloid cell expression of CD200R is modulated in active TB disease and regulates *Mycobacterium tuberculosis* infection in a biomimetic model

**DOI:** 10.3389/fimmu.2024.1360412

**Published:** 2024-04-30

**Authors:** Mohamed Ahmed, Liku B. Tezera, Nicholas Herbert, Mark Chambers, Michaela T. Reichmann, Kievershen Nargan, Henrik Kloverpris, Farina Karim, Mbali Hlatshwayo, Rajhmun Madensein, Munir Habesh, Monjural Hoque, Adrie J.C. Steyn, Paul T. Elkington, Alasdair J. Leslie

**Affiliations:** ^1^ Africa Health Research Institute, Durban, South Africa; ^2^ College of Health Sciences, School of Laboratory Medicine & Medical Sciences, University of KwaZulu Natal, Durban, South Africa; ^3^ NIHR Biomedical Research Centre, School of Clinical and Experimental Sciences, Faculty of Medicine, University of Southampton, Southampton, United Kingdom; ^4^ Institute for Life Sciences, University of Southampton, Southampton, United Kingdom; ^5^ Department of Immunology and Microbiology, University of Copenhagen, Copenhagen, Denmark; ^6^ Department of Infection and Immunity, University College London, London, United Kingdom; ^7^ Department of Cardiothoracic Surgery, Nelson Mandela School of Medicine, University of KwaZulu-Natal, Durban, South Africa; ^8^ Kwadabeka Community Health Care Centre, Kwadabeka, South Africa; ^9^ Department of Microbiology, University of Alabama at Birmingham, Birmingham, AL, United States

**Keywords:** tuberculosis, pulmonary infection, immune checkpoint, CD200 receptor, innate immunity

## Abstract

A robust immune response is required for resistance to pulmonary tuberculosis (TB), the primary disease caused by *Mycobacterium tuberculosis* (*Mtb*). However, pharmaceutical inhibition of T cell immune checkpoint molecules can result in the rapid development of active disease in latently infected individuals, indicating the importance of T cell immune regulation. In this study, we investigated the potential role of CD200R during *Mtb* infection, a key immune checkpoint for myeloid cells. Expression of CD200R was consistently downregulated on CD14^+^ monocytes in the blood of subjects with active TB compared to healthy controls, suggesting potential modulation of this important anti-inflammatory pathway. In homogenized TB-diseased lung tissue, CD200R expression was highly variable on monocytes and CD11b^+^HLA-DR^+^ macrophages but tended to be lowest in the most diseased lung tissue sections. This observation was confirmed by fluorescent microscopy, which showed the expression of CD200R on CD68^+^ macrophages surrounding TB lung granuloma and found expression levels tended to be lower in macrophages closest to the granuloma core and inversely correlated with lesion size. Antibody blockade of CD200R in a biomimetic 3D granuloma-like tissue culture system led to significantly increased *Mtb* growth. In addition, *Mtb* infection in this system reduced gene expression of CD200R. These findings indicate that regulation of myeloid cells via CD200R is likely to play an important part in the immune response to TB and may represent a potential target for novel therapeutic intervention.

## Introduction

Despite effective drug therapy, tuberculosis (TB) remains a leading cause of death globally. Annually, approximately 10 million fall ill, and there were 1.6 million deaths in 2021 (1). The causative agent of human TB, *Mycobacterium tuberculosis* (*Mtb*), is an ancient pathogen that primarily affects the lungs, although dissemination to extrapulmonary sites is possible (2–5). Protective TB immunity depends on the vigorous induction of T cells and innate effectors such as macrophages and neutrophils (6). However, chronic inflammation due to persistent *Mtb* infection is a key driver of immunopathology manifesting as cavitary disease in TB patients (7, 8). Cavities facilitate the expulsion of aerosolized bacilli via the airways for transmission to a new host, thereby completing the bacterial life cycle. A considerable body of evidence indicates that negative immune regulation of the T cell response to *Mtb* is critical to preventing excessive inflammation and maintaining lung health during *Mtb* infection (9).

Immune checkpoint (IC) molecules are inhibitory receptors that induce immunosuppressive signaling pathways (10). Primarily expressed by T cells, ICs play a key role in maintaining self-tolerance and limiting immunopathology. One of the most well-studied of these is programmed cell death protein 1 (PD-1), primarily due to its exploitation in novel cancer therapies. Inhibition of PD-1 or its receptors results in more robust Th1 responses and has proven a successful therapeutic approach to treating multiple cancers (11). However, numerous clinical case reports and pharmacovigilance analyses have shown that blockade of PD-1 can lead to reactivation of latent TB (9, 12). This is mainly thought to be due to a reinvigorated T-cell immune responses driving immunopathology and progression of previously stable TB lesions (13–15). Likewise, PD-1 deficiency in mice significantly exacerbated *Mtb* infection and was rapidly lethal (16, 17). Therefore, unrestrained T cell responses appear to undermine effective TB immunity.

Monocytes are recruited from the blood at the site of *Mtb* infection and can differentiate into dendritic cells and macrophages (18). In mouse models, dendritic cells and macrophages are the primary sites of *Mtb* replication during later stages of infection (19). Importantly, *Mtb* has been shown to modulate monocyte surface markers (20), and several of these, such as CD64, CD163, and CD14, have been identified as putative biomarkers of active TB disease (21). Resident alveolar macrophages (AMs) constitute the lung’s first line of immune cell defense against Mtb (22). To limit harmful immune responses that could impair lung function, AMs maintain an inherently anti-inflammatory phenotype characterized by constitutive expression of inhibitory molecules, including the CD200 receptor (CD200R) (23, 24). In the airway, the interaction between CD200R on AMs and CD200 expressed by alveolar epithelial cells induces a hyporesponsive phenotype (23). Mice deficient for the inhibitory CD200-CD200R pathway are highly susceptible to influenza and *F. tularensis infection* due to excessive inflammation (25, 26). Conversely, engagement of the CD200-CD200R axis mitigates lung inflammation in asthma (27). Patients suffering from the systemic inflammatory syndrome sarcoidosis exhibited decreased expression of CD200R on circulating monocytes compared to healthy controls (28). In light of these observations, we questioned whether the CD200-CD200R axis played a role in the immune response to *Mtb* infection.

## Methods

### Study participants

Whole blood was collected from participants with microbiologically confirmed pulmonary TB (n=15) before treatment initiation, enrolled in the CUBS study (BREC# BE022/13). Control samples (n=15) were collected from HIV-uninfected individuals without signs and symptoms of TB enrolled in the HINTS study (BREC# BE083/18). Study subjects were not tested for Mtb infection by interferon gamma release assay (IGRA) or other means. However, the force of infection in Durban is extremely high and approximately 60% of HIV uninfected participants in a recent study from a similar Durban Cohort were found to be IGRA positive (29). Thus, this group is highly likely to contain both IGRA+ve and IGRA-ve participants. TB-diseased lung was received through the AHRI lung study (BREC# BE019/13), as described previously (30, 31) which obtains surgically resected lung tissue removed to treat TB sequelae. For each resection, 3 tissue blocks were removed by the operating surgeon and corresponding to their assessment of the most and least diseased sections of the removed tissue.

### PBMC isolation

Peripheral blood mononuclear cells (PBMCs) were isolated by density gradient centrifugation using Histopaque (Sigma). Whole blood was carefully layered onto Histopaque, followed by centrifugation with brakes off. The buffy coat layer was isolated, washed, and resuspended in freezing media (90% FBS, 10% DMSO). Before staining, cells were thawed and resuspended in prewarmed RPMI 1640. Following washing with PBS, cells were rested for 2 hours at 37 ˚C, 5% CO_2_.

### Lung tissue homogenization

The lung tissue was processed as described (31). Briefly, tissue was dissected into small pieces and mixed with collagenase (Sigma-Aldrich) DNase 1 (Sigma-Aldrich) in RPMI (Sigma-Aldrich) with 10% FBS (Hyclone) for 30 minutes and then mechanically digested at room temperature using the Gentle MACS system (Miltenyi Biotec) followed by agitation at 37°C for 30 minutes. This mechanical digestion was repeated, and the subsequent suspension was filtered using the 70mm (Corning) cell strainer, followed by red blood cell lysis. Cells were then stained for flow cytometry as described below.

### Immunophenotyping

PBMCs were pelleted, washed again, and incubated with 25 μL of an antibody cocktail for 1 hour at 4˚C. Surface markers included CD45-HV500 (clone: Hi30, BD), CD14-BV711 (clone: M5E2, Biolegend), CD3-Qdot800 (clone: OKT3, Biolegend), CD200R-PE (clone: OX108, Biolegend) and LIVE/DEAD™ Fixable Near-IR Dead Cell. Lung cells were stained for 20 minutes at room temperature in the dark. The antibody cocktail for the lung homogenates contained CD45-APC (clone: Hi30, Biolegend), CD11b-PE-Cy7 (clone: ICRF44, BD), CD3-APC-Cy7 (clone: OKT3, Biolegend), CD200R-PE (clone: OX108, Biolegend) and Invitrogen LIVE/DEAD™ Aqua fluorescent reactive dye. After a final washing step, the cells were fixed in 2% PFA until acquisition. Data was acquired using a BD Aria Fusion cytometer and analyzed using FlowJo Software v.9.9.

### Immunofluorescent imaging

The multiplex fluorescent immunohistochemistry (F-IHC) experiments were performed using the Opal 4-Color Manual IHC kit (Akoya Biosciences) according to the manufacturer’s instructions. Briefly, formalin (4%) fixed lung tissue samples were embedded in paraffin, and 4 μm sections were obtained on glass slides. These sections were deparaffinized and incubated with the following primary antibodies: CD68 (clone: KP1, Dako, 1:200), CD3 (clone: Sp7, Abcam, 1:250) and CD200R (clone: polyclonal, Abcam, 1:1000). Opal fluorophore FITC (Opal 520) was used for CD68, Texas-Red (Opal 570) was used for CD3 and Cy5 (Opal 690) was used for CD200R, for signal generation. DAPI was used as the nuclear counterstain. After this, sections were mounted with Opal mounting oil, cover-slipped, and the edges sealed. The slides were stored at 4°C until images were acquired (within 24 hours). Images were acquired on a Zeiss Axio Observer Z1 inverted microscope (Olympus) and analyzed with TissueFAXS software (TissueGnostics).

### Bacterial culture

Bioluminescent *Mtb* H37Rv was grown in Middlebrook 7H9 broth (Difco) (supplemented with 10% OADC, 0.2% glycerol, and 0.02% Tween 80) containing 25 μg/mL kanamycin. This bioluminescent *Mtb* strain expresses the luxABCDE operon from *P. luminescens* (32, 33). The system allows for constitutive emittance of luminescence, without the use of substrate. Cultures at 1 x 10^8^ CFU/mL *Mtb* (OD = 0.6) were used for all experiments at a multiplicity of infection of 0.1.

### 3D microsphere generation

The generation of microspheres is described in Tezera et al. (33). Briefly, PBMCs were infected with bioluminescent *Mtb* overnight and recovered the following day. At this stage, cells were incubated with anti-CD200R or isotype for 1 hour at 4˚C, after which the cell suspension was mixed with the alginate-collagen matrix. This mixture was injected into the bead generator (Nisco) to encapsulate the cells into microspheres in a gelling bath containing CaCl_2_. Next, the microspheres were washed with HBSS (Gibco) and resuspended in RPMI 1640 (Gibco) containing 10% human AB serum, 1% ampicillin, and 25 mg/mL kanamycin. Spheres were transferred into 24-well white plates and incubated at 37˚C, 5% CO_2_. Growth of *Mtb* within microspheres was measured longitudinally by luminescence with a GloMax 20/20 Luminometer (Promega). Each experimental condition was performed in triplicates.

### RNA sequencing

RNA sequencing analysis conducted on microspheres and lung lymph nodes is described in detail by Reichmann et al. (34). Briefly, sequencing was performed by GENEWIZ. Samples were sequenced using Illumina HiSeq; a configuration of 2 × 150 base pairs per lane was used, and a mean average of 31 million paired end reads generated. Alignment was performed using kallisto software (version 43.1), with sequence based bias correction. Differential gene analysis was performed using limma with its voomWithQualityWeights function (version 3.38.3). All analyses were performed in R environment.

### Cell viability assay

Microspheres were incubated in 96-well plates at 37°C. Cell viability was analysed on day 7 using the CytoTox-Glo Cytotoxicity Assay (Promega), which detects cellular necrosis in microspheres. Luminescence was analysed by GloMax Discover (Promega). To provide the denominator, total cell death was caused by digitonin after the initial measurement.

### TNF-α ELISA

TNF-α was quantified using a sandwich ELISA kit from ImmunoTools (Friesoythe, Germany) on supernatants collected on day 7 according to the manufacturer’s instructions. The typical standard curve range is 20-2000 pg/ml, and the lower limit of detection is 22 pg/ml.

### Statistical analysis

Analysis was performed in GraphPad Prism v10. Kruskal-Wallis test with Dunn’s multiple comparison test was performed for groups of 3 or more groups. For the flow cytometric analysis of clinical samples, data were analyzed using the Mann-Whitney test. Correlation between granuloma size and CD200R expression was analyzed by linear regression.

## Results

**Figure 1:**
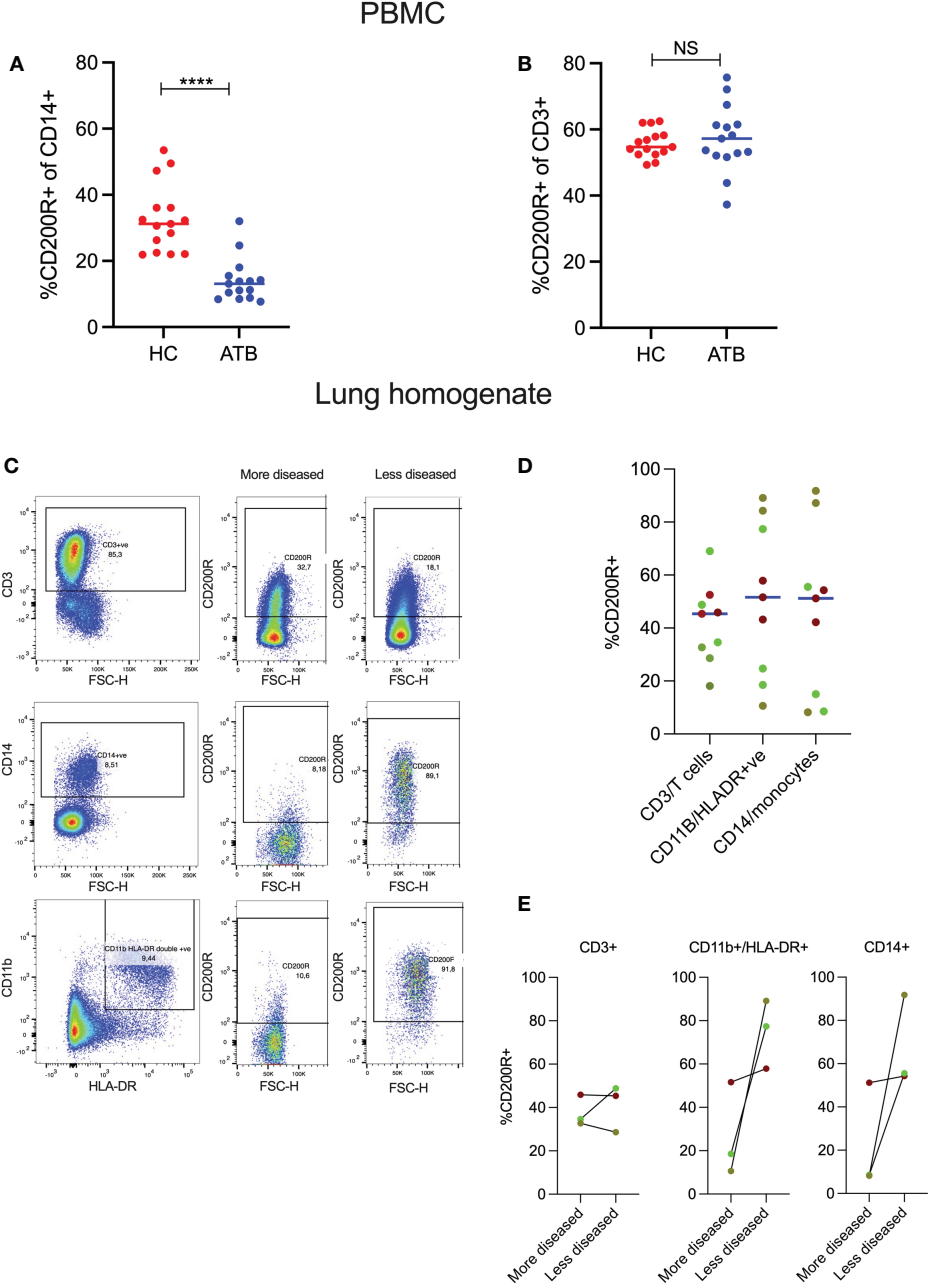
CD200R expression in blood and TB-affected lung tissue. PBMCs were isolated from healthy controls (HC) and active TB patients (ATB), *n*=15 for each group. Expression of CD200R on monocytes (CD14^+^) was significantly reduced in the blood of active TB patients compared to healthy controls **(A)**. In contrast, no differences in CD200R expression were observed on T cells (CD3^+^) between the two groups **(B)**. Representative flow cytometry plots of 2 TB lung tissue samples resected from different areas of the same lung **(C)**. CD200R expression on T cells, macrophages, and monocytes in 3 TB-diseased lungs (3 samples per patient) as assessed in samples with varying degrees of pathology **(D)**. Expression of CD200R expression on T cells, macrophages, and monocytes within a single lung comparing areas differing pathology **(E)**. The dots of the same color indicate individual tissue samples from the same lung. Mann-Whitney test was used for the statistical analysis. ****p < 0.0001; NS, not significant.

To determine CD200R expression, we analyzed PBMCs from participants with active TB (ATB) and healthy controls (HC) by flow cytometry (**Supplementary Figure 1**). ATB was associated with significantly lower frequency of CD200R-expressing CD14^+^ monocytes (median = 13.1%) compared to HC (median = 31.2%, P = <0.0001; **Figure 1A**). This is consistent with an enhanced pro-inflammatory phenotype of circulating monocytes from TB patients (35). In contrast, there were no differences in the frequency of CD200R-expressing CD3^+^ T cells in ATB (median = 57.20%) compared to HC (median = 57.7%, P = 0.78) (**Figure 1B**).

Next, to determine whether CD200R expression was also reduced on myeloid cells in the lung, we performed flow cytometry on lung tissue sections isolated from 3 individuals (3 tissue samples per patient) with TB disease undergoing surgical resection to treat TB sequelae (31). The 3 tissue blocks were selected by the operating surgeon based to include the most and least diseased sections of the lung tissue removed. It is important to note, however, that this is a crude categorization as the extent of disease varies between subjects and the selection of tissue blocks is based on macroscopic appearance. All tissue blocks were homogenized to generate a single cell suspension and CD200R expression determined by flow cytometry as above. As CD14^+^ monocytes typically mature into macrophages or other myeloid subsets when they migrate into inflamed tissue, CD200R expression levels were assessed on CD14^+^ monocytes and CD11b^+^HLA-DR^+^ cells, consistent with a macrophage phenotype. **Figure 1C** shows CD200R expression on two tissue blocks from a TB-diseased lung corresponding to the most and least diseased tissue sections as defined by the surgeon. In the more diseased tissue block, CD200R is expressed on CD3^+^ T-cells but only on a very small fraction (<10%) of CD14^+^ monocytes or CD11b^+^HLA-DR^+^ myeloid cells (**Figure 1C**). In contrast, in the less diseased tissue block from the same lung, most CD14^+^ and CD11b^+^HLA-DR^+^ cells expressed CD200R. This pattern of expression was reflected in one of the two other lungs studied (bright green dots), while a third lung (red dots) had a similar frequency of CD200R-expressing myeloid cells in all tissue blocks analyzed (**Figure 1D**). Moreover, in two of the lungs, CD200R expression on CD11b^+^HLA-DR^+^ cells as well as CD14^+^ cells were markedly increased in sections exhibiting less pathology (**Figure 1E**). These data suggest that CD200R expression on myeloid cells is heterogenous in TB diseased lung tissue and tends to be lower in tissue sections with more overt disease.

**Figure 2:**
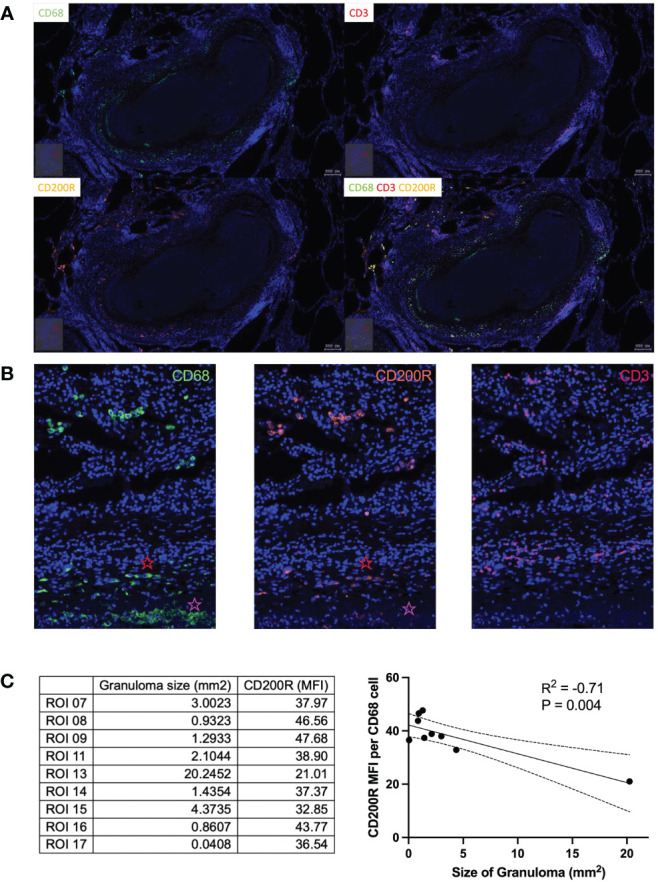
CD200R expression in TB lung tissue. **(A)** In lung tissue resected from a TB patient, CD200R was highly expressed in the cuff of granulomas. **(B)** CD200R expression was mainly exhibited on macrophages, as evidenced by the colocalization of CD200R and CD68 staining. Red star = distal from granuloma core, pink star = proximal to granuloma core. **(C)** CD200R expression was analyzed against granuloma size. By linear regression the size of granulomas was negatively correlated (R^2 = ^0.71) with CD200R expression on CD68^+^ macrophages. ROI, region of interest; MFI, mean fluorescence intensity.

To investigate this further, we next determined the relationship between CD200R and TB lesions within the lung by fluorescence microscopy. A lung tissue section containing multiple TB lesions was scanned and analyzed using the TissueFAX software to extract single-cell expression data. The cellular composition of granuloma displayed a conventional CD68^+^ macrophage cuff around an acellular core, surrounded by an outer zone of CD3^+^ T cells (**Figure 2A**). In contrast to the circulating monocytes (**Figure 1A**), CD200R was highly expressed on CD68^+^ cells located on the periphery of granulomas (**Figure 2A**). As shown in **Figure 2B**, CD200R was expressed on CD68^+^ macrophages both distal and proximal to the granuloma core. As expected, macrophages proximal to the granuloma center (indicated with a red star) displayed a more epithelioid morphology, consistent with granuloma-associated macrophages. Interestingly, CD200R expression was less consistent within these macrophages, particularly near the granuloma core (indicated with a pink star). To examine this further, nine granulomas within the tissue section were scanned, and CD200R expression levels were quantified on CD68^+^ macrophages within each lesion (**Supplementary Figure 2**). In addition, TissueFAX software allows the measurement of area by demarcation, so granuloma size was determined this way. This revealed a significant negative association between lesion size and macrophages CD200R expression level (**Figure 2C**). Taken together, these data suggest that CD200R is expressed on granuloma-associated macrophages within the lung, but the expression level is variable and may be reduced in cells closest to the granuloma core and in the larger granuloma.

**Figure 3:**
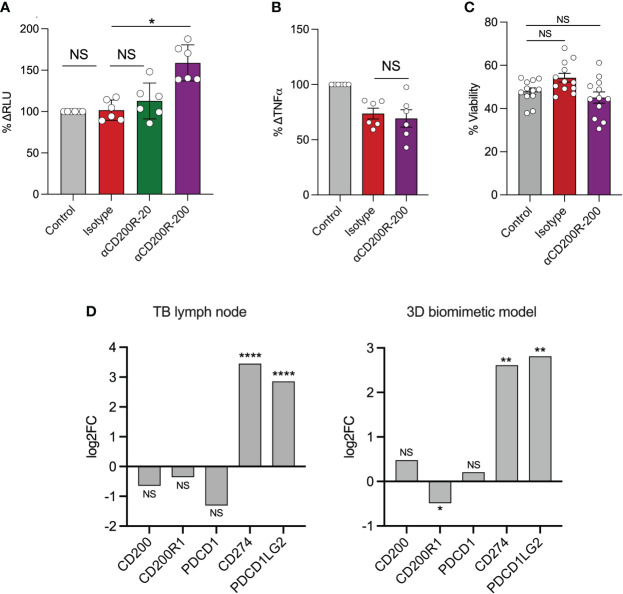
Inhibition of CD200R accelerates *Mtb* growth in a 3D cellular model. In a 3D biomimetic model of TB, antibody-mediated inhibition of CD200R led to a significantly increased *Mtb* burden at day 7 as measured by relative luminescent units (RLU) as shown as percenta **(A)**. This was only observed at the highest concentration (μg/mL) of blocking antibody used. **(B)** CD200R blockade did not result in an increase in TNF-α secretion from infected microspheres. **(C)** Likewise, cell viability was not affected by CD200R blockade. **(D)** RNA sequencing showed a non-significant decrease in *CD200* and *CD200R1* expression in TB lymph nodes compared to uninfected control. In contrast *CD200R1* in the 3D model was significantly decreased compared to uninfected control. Data are shown as mean ± SEM of at least two independent experiments. Kruskal-Wallis test with Dunn’s multiple comparison test was used for the statistical analysis. *p < 0.05, **p < 0.01, ****p < 0.0001. NS, not significant.

In light of these results, we tested the effect of CD200R inhibition on bacterial control using a 3D granuloma model (33). For this, PBMCs were infected with bioluminescent *Mtb* and suspended in an alginate-collagen matrix. This mixture was passed through a cell encapsulator to generate microspheres, allowing the cells to aggregate in 3D, with collagen providing an extracellular matrix and thus mimicking the lung tissue environment. Previously, we demonstrated in this model that PD-1 blockade accelerated *Mtb* growth, driven by an exacerbation of the TNF-α response (36). The addition of 200 μg/mL of CD200R blocking antibody resulted in a significant increase in bacterial growth, as shown by an increase in the luminescent signal at day 7 (**Figure 3A**), previously shown to correlate closely with *Mtb* burden (37). Addition of 20 μg/mL of anti-CD200R or an isotype control did not affect *Mtb* growth. In contrast to PD-1 inhibition, which greatly increased TNF-a secretion, CD200R blockade led to a decrease in TNF-a levels, although this did not reach statistical significance (**Figure 3B**). Cell viability was assessed to test whether the increase in *Mtb* growth upon CD200R blockade was due to cell death. Incubation with either the isotype control or anti-CD200R, however, did not significantly impact cell viability compared to untreated controls (**Figure 3C**). Finally, we examined CD200R gene expression in published data generated by our groups from human TB granuloma and the 3D model (34). Bulk RNA sequence data from laser captured untreated TB granuloma identified in lymph nodes showed a slight reduction in the expression of *CD200* and *CD200R* compared to uninfected lymph nodes, although the difference did not reach statistical significance (**Figure 3D**). Interestingly, we observed a similar decrease in the expression of *PDCD1* (gene encoding PD-1) but the expression of the genes encoding its two ligands *CD274* (PD-L1) and *PDCD1LG2* (PD-L2), were highly increased. In the 3D model, we observed a similar and statistically significant reduction of *CD200R1* expression during *Mtb* infection compared to uninfected spheres, but no change in *CD200* expression (**Figure 3D**). As in the lymph nodes, *CD274* and *PDCD1LG2* were highly increased, consistently with the overall transcriptional similarity between *in vivo* TB granuloma and the 3D model system (34). It is worth noting that highly elevated PDL1 protein expression has been observed in human TB lung granuloma (38). These data are consistent with reduction in of CD200R expression observed on circulating monocytes in TB patients (**Figure 1A**) and with reduced CD200R expression in progressive lung granuloma (**Figure 2B, C**).

## Discussion

Here, for the first time to our knowledge, we show that CD200R expression is significantly and consistently decreased in circulating CD14^+^ monocytes from TB patients compared to healthy controls. In TB lung tissue, CD200R expression was variable across the diseased lung on CD14^+^ monocytes and CD11b^+^HLA-DR^+^ macrophages, and some tissue sections, obtained from the most diseased parts of the lung, contained little or no CD200R^+^ myeloid cells. Histological analysis revealed that CD200R was expressed on CD68^+^ macrophages associated with granuloma. However, in TB-diseased lungs, CD200R expression levels appeared lower on macrophages closest to the necrotic core. In addition, analysis of all 9 TB lesions in this tissue section suggests that expression levels of CD200R decreased as lesion size increased. Although significant, we are cautious in extrapolating this data, as it represents a single slide from a single participant and includes an outlying data point from only a single larger lesion. It will, therefore, be important to confirm these findings in additional samples. The lack of uninfected controls for the lung homogenates as well as the lung tissue section is a limitation of this study. Nevertheless, it is clear that CD200R expression levels on circulating monocytes are modulated in TB disease, strongly support a potential role for altered CD200R signaling within the lung associating with TB disease. This hypothesis is supported by independent experiments showing that the blockade of CD200R significantly increased *Mtb* growth in the 3D-granuloma model; and that *Mtb* infection in the 3D model induces reduced gene expression of *CD200R1*. Therefore, these data indicate that, like T-cells, regulation of myeloid immune responses in the lung is likely to play an important part in limiting immunopathology in TB.

The majority of TB in adults develops in immune-experienced host and is termed post-primary disease. In post-primary TB, pathology can develop as a function of immunodeficiency, as in the case of progressive HIV or hyperinflammation (39). This later mechanism is exemplified by clinical and experimental studies showing that the loss of negative regulation of T-cells via PD-1/PDL-1 accelerates the onset of TB disease (9, 12, 13). Equivalent data is unavailable for CD200R, but LPS-induced lung inflammation is exacerbated in CD200-deficient rats as a result of greater alveolar macrophage activation (40). Likewise, mice lacking CD200 have an exaggerated response to influenza infection, leading to slower resolution of infection and greater lung damage (25). Interestingly, several viruses have evolved to express CD200-like proteins on the surface of infected cells, which interact with CD200R and inhibit killing (41). Thus, it is not unexpected that the CD200/CD200R axis might play an important role in modulating TB immunopathology. Like PD-1/PDL-1, CD200R has aroused interest as a potential target in the treatment of tumors (42). CD200 is highly expressed on tumor cells, impairs antitumor immune responses, and is associated with poor prognosis. Although the blockade of CD200 has been shown to promote antitumor immunity, definitive clinical trial data on efficacy is still lacking (41). If CD200/CD200R blocking agents proceed to further clinical trials, however, it will be important to look for early signals of TB reactivation. In conclusion, these data show that CD200R expression is modulated during active TB in humans, on both circulating monocytes and myeloid cells within TB-diseased lungs, and is likely to play an important role in balancing the inflammatory responses during *Mtb* infection.

## Data availability statement

The original contributions presented in the study are included in the article/**Supplementary Materials**, further inquiries can be directed to the corresponding author/s.

## Ethics statement

The studies involving humans were approved by Biomedical Research Ethics Committee (UKZN). The studies were conducted in accordance with the local legislation and institutional requirements. The participants provided their written informed consent to participate in this study.

## Author contributions

MA: Data curation, Formal analysis, Investigation, Methodology, Software, Validation, Visualization, Writing – original draft, Writing – review & editing, Conceptualization. LT: Conceptualization, Data curation, Formal Analysis, Investigation, Methodology, Software, Supervision, Validation, Visualization, Writing – original draft, Writing – review & editing. NH: Formal Analysis, Methodology, Resources, Software, Writing – review & editing. MC: Data curation, Formal analysis, Methodology, Resources, Software, Writing – review & editing. MR: Writing - review & editing, Formal analysis. KN: Methodology, Resources, Software, Writing – review & editing. HK: Funding acquisition, Investigation, Project administration, Resources, Supervision, Writing – review & editing. FK: Funding acquisition, Methodology, Project administration, Resources, Supervision, Validation, Writing – review & editing. MbH: Methodology, Resources, Supervision, Writing – review & editing. RM: Methodology, Resources, Supervision, Writing – review & editing. MuH: Methodology, Resources, Supervision, Writing – review & editing. MoH: Methodology, Project administration, Resources, Supervision, Writing – review & editing. AS: Conceptualization, Funding acquisition, Investigation, Methodology, Project administration, Resources, Supervision, Writing – review & editing. PE: Conceptualization, Funding acquisition, Investigation, Methodology, Project administration, Resources, Supervision, Writing – review & editing. AL: Conceptualization, Data curation, Formal analysis, Funding acquisition, Investigation, Methodology, Project administration, Resources, Supervision, Writing – original draft, Writing – review & editing.

## Supplementary material

Supplementary Figure 1 Gating strategy to analyze CD200R expression on peripheral monocytes and T cells.

Supplementary Figure 2 TB-diseased lung tissue section with multiple granulomas. Lesion size indicated (mm^2^).
