# Supplementary figures and images for: Myeloid cell expression of CD200R is modulated in active TB disease and regulates Mycobacterium tuberculosis infection in a biomimetic model
Source: Front Immunol. 2024 Apr 30;15:1360412. doi: 10.3389/fimmu.2024.1360412 (PMC11091283; doi:10.3389/fimmu.2024.1360412)

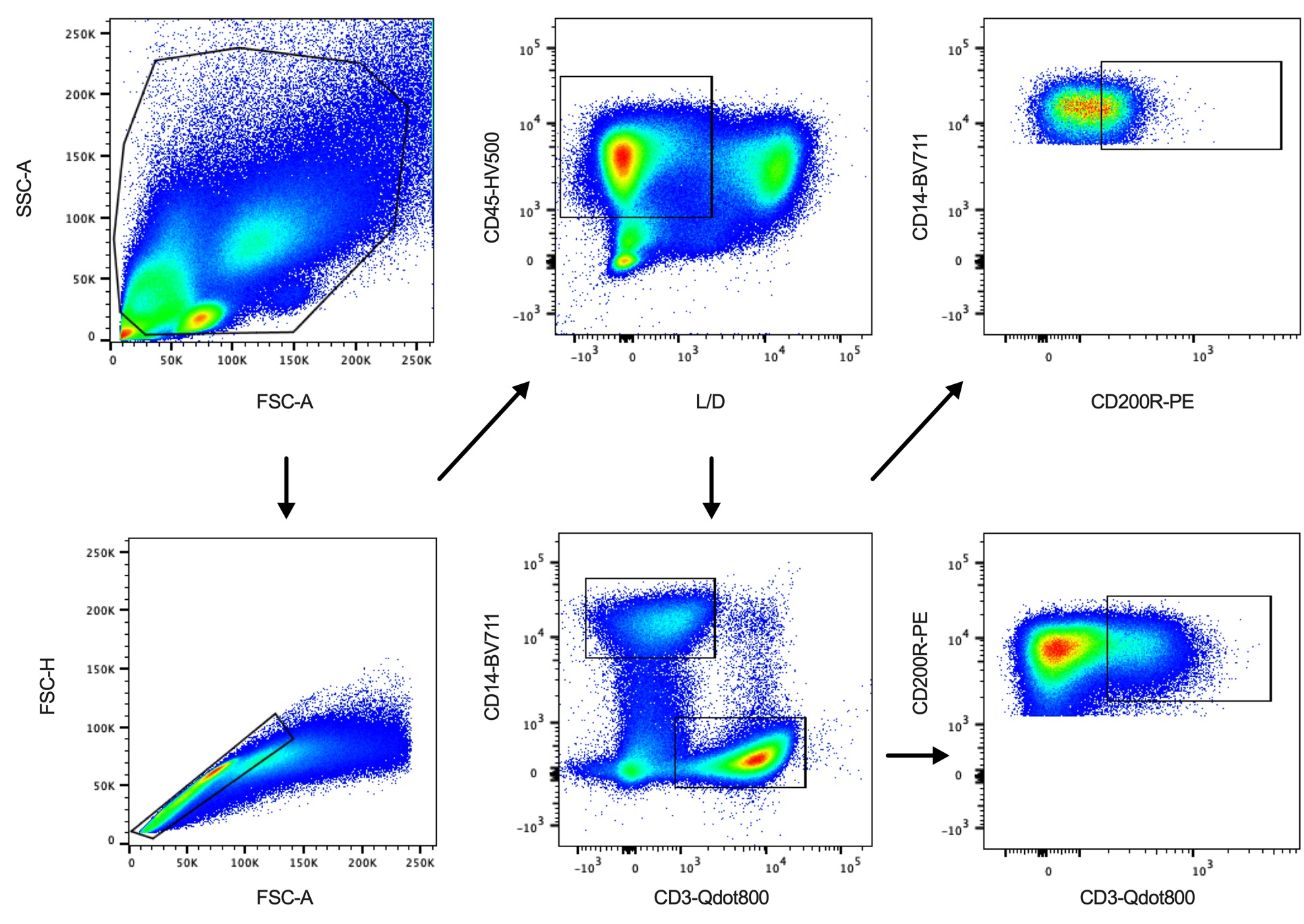

Supplement: Supplementary Figure 1 — Gating strategy to analyze CD200R expression on peripheral monocytes and T cells. [file Image_1.jpeg]

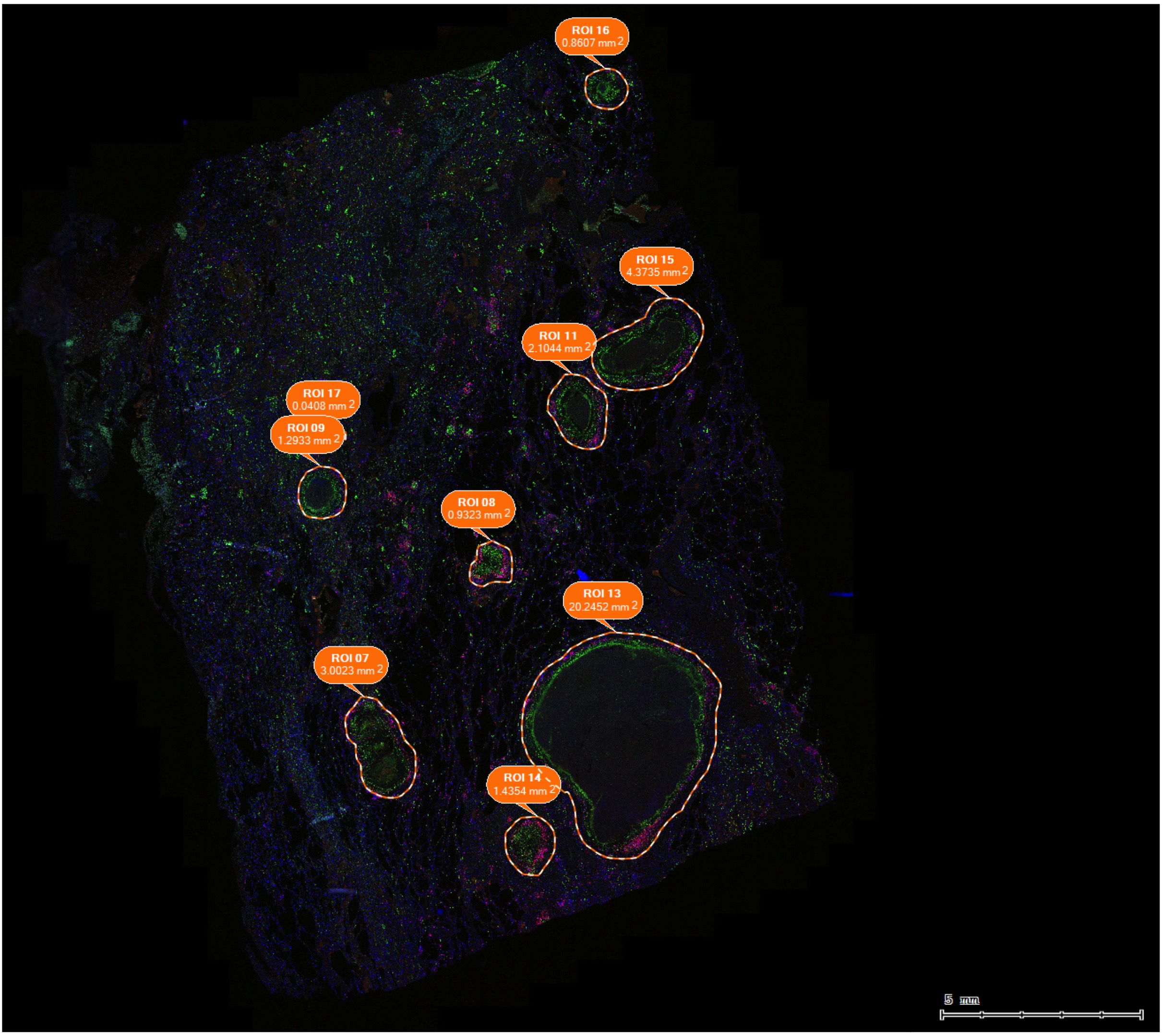

Supplement: Supplementary Figure 2 — TB-diseased lung tissue section with multiple granulomas. Lesion size indicated (mm2). [file Image_2.jpeg]
